# HelexKids: A word frequency database for Greek and Cypriot primary school children

**DOI:** 10.3758/s13428-015-0698-5

**Published:** 2016-01-28

**Authors:** Aris R. Terzopoulos, Lynne G. Duncan, Mark A. J. Wilson, Georgia Z. Niolaki, Jackie Masterson

**Affiliations:** 1 Psychology, School of Social Sciences, University of Dundee, Nethergate, DD1 4HN Dundee, UK; 2 Dundee, UK; 3 School of Psychology, Behavioral and Social Sciences, Coventry University, Coventry, UK; 4 Department of Psychology and Human Development, Institute of Education, University College London, London, UK

**Keywords:** Word database, Greek language, Children, Frequency, Contextual diversity

## Abstract

In this article, we introduce HelexKids, an online written-word database for Greek-speaking children in primary education (Grades 1 to 6). The database is organized on a grade-by-grade basis, and on a cumulative basis by combining Grade 1 with Grades 2 to 6. It provides values for Zipf, frequency per million, dispersion, estimated word frequency per million, standard word frequency, contextual diversity, orthographic Levenshtein distance, and lemma frequency. These values are derived from 116 textbooks used in primary education in Greece and Cyprus, producing a total of 68,692 different word types. HelexKids was developed to assist researchers in studying language development, educators in selecting age-appropriate items for teaching, as well as writers and authors of educational books for Greek/Cypriot children. The database is open access and can be searched online at www.helexkids.org.

This article presents HelexKids [*Helex* stems from Ελλάς (*Hellas*) = Greece and λέξη (*lexi*) = word], the first psycholinguistic database that provides grade-based written-word frequency (Grades 1 to 6) for Greek and Greek-Cypriot primary school children. The word corpus contains 68,692 different nonlemmatized word types, taken from a total of 1,355,265 tokens from 116 textbooks used in primary education in Greece. This online database of words from children’s texts provides a Web facility for developmental research in the Greek language, as well as a resource for educators involved in Greek education.

## Psycholinguistic background

Psycholinguistic word databases have been developed mainly to contribute to cognitive research with adults. Computerized adult databases have been available for many years for a variety of languages in Europe and the USA [e.g., the Brown corpus (Kučera & Francis, 1967), for American English; the British National Corpus (2007), for British English; CELEX (Baayen, Piepenbrock, & Gulikers, 1995), for English, Dutch, and German; the Hellenic National Corpus (2009; hereafter, HNC), for Greek; and Lexique (New, Pallier, Brysbaert, & Ferrand, 2004), for French—to mention only a few, widely used examples]. Lately, large-scale databases have been constructed from the Internet (e.g., HAL; Lund & Burgess, 1996), from Google (Brants & Franz, 2006), or from television subtitles (e.g., SUBTLEX). The latter have been found to explain more variance in adult lexical decision times than do previous databases based on printed material. Subtitle-based corpora have been made available for a number of languages [e.g., SUBTLEX (New, Brysbaert, Véronis, & Pallier, 2007), for French; SUBTLEX-US (Brysbaert & New, 2009), for American English; SUBTLEX-NL (Keuleers & Brysbaert, 2010), for Dutch; SUBTLEX-GR (Dimitropoulou, Duñabeitia, Avilés, Corral, & Carreiras, 2010), for Greek; SUBTLEC-CH (Cai & Brysbaert, 2010), for Chinese; SUBTLEX–ESP (Cuetos, Glez-Nosti, Barbón, & Brysbaert, 2011), for Spanish; SUBTLEX-DE (Brysbaert et al., 2011), for German; SUBTLEX-UK (van Heuven, Mandera, Keuleers, & Brysbaert, 2014), for British English; and SUBTLEX-PT (Soares et al., 2015), for Portuguese].

A core component of adult databases is objective frequency, which equates to the count of word occurrences in textbooks, subtitles, or Internet-based texts and has proved to be one of the most important word statistics in cognitive research. It is well established that more variance is explained by frequency than by semantic or lexical variables (e.g., number of meanings, word category, neighborhood) in lexical decision and, to a lesser extent, in speeded word naming, in which word onset and length also play a significant role (Baayen, Feldman, & Schreuder, 2006; Balota, Cortese, Sergent-Marshall, Spieler, & Yap, 2004; Brysbaert et al., 2011).

The necessity of including objective frequency measures in experimental research stems also from the consistently differential effects that high- and low-frequency words have on item processing, production, and recognition. High-frequency words facilitate target recognition in lexical decision tasks, whereas the opposite is observed for low-frequency words (Mason, 1976; Monsell, 1991; van Heuven, Mandera, Keuleers, & Brysbaert, 2014). This effect was observed for the reaction times (RTs) in both the English Lexicon Project (Balota et al., 2007) and the British Lexicon Project (Keuleers, Lacey, Rastle, & Brysbaert, 2012). Furthermore, the same pattern of faster RTs for high-frequency words was observed by Duyck, Vanderelst, Desmet, and Hartsuiker (2008) for Dutch–English bilinguals when recognizing words in their second language. Other differential effects include observations that low-frequency words produce more phonological errors in speech than do high-frequency words (Stemberger & MacWhinney, 1986), that low-frequency words are recognized better in recognition memory experiments than high-frequency words (known as the *mirror effect*; Shepard, 1967; Steyvers & Malmberg, 2003), and that pictures are named faster when they correspond to high- rather than low-frequency words (Jescheniak & Levelt, 1994).

Measures of subjective frequency (e.g., Balota, Pilotti, & Cortese, 2001) and age of acquisition (AoA; e.g., Cortese & Khanna, 2007) were only able to explain additional naming or lexical decision variance when the objective frequency values used as predictors in the same analysis were taken from less reliable databases, such as the Kučera–Francis frequency norms (Brysbaert & Cortese, 2011). Although this was less true of AoA ratings, there is uncertainty over whether AoA is as important a predictor in transparent orthographies as it is in opaque ones (Burani, Arduino, & Barca, 2007).

More recent research has explored the contribution of an alternative conceptualization of frequency in lexical processing—namely, contextual diversity (CD), which is an index of the number of different contexts in which a word occurs. The more contexts that a word appears in, the greater the probability of that word being encountered by a reader. In a thorough investigation of the role of CD in recognition times, Adelman, Brown, and Quesada (2006) found that CD accounted for more variance in lexical decision and naming than did word frequency. CD was also observed to explain more variance than did frequency in lexical decision performance when using values obtained from SUBTLEX databases (Brysbaert & New, 2009; Dimitropoulou et al., 2010; Soares et al., 2015). Baayen (2010) found that CD was one of the most significant components, along with morphological and syntactic family size, in the prediction of lexical decision latencies. The explanatory value of CD has also been investigated using an eyetracking paradigm (Plummer, Perea, & Rayner, 2014) in which skilled readers silently read sentences and then had to answer true–false comprehension questions. In this case, CD but not frequency was found to significantly affect fixation and gaze durations.

### Written frequency databases for developmental research

Developmental psycholinguistic databases tend to be compiled using children’s textbooks, as this type of corpora is recognized as possessing several essential qualities for experimental research—namely, that the information is up-to-date, large in scale, and form-appropriate for the purpose of study (Brysbaert et al., 2011). The availability of databases based on a wide range of children’s textbooks that are actually read and used in schools enables researchers to extract accurate figures for reliable testing of lexical processing among children, to parallel the experimental work on adults. This is particularly relevant now that investigations of developing readers are increasingly complemented by online methodologies that demand a high degree of measurement precision, such as computerized masked-priming tasks (e.g., Castles, Davis, Cavalot, & Forster, 2007), neuroimaging (e.g., Conant, Liebenthal, Desai, & Binder, 2014; Jasinska & Petitto, 2014), or eyetracking (e.g., Rau, Moeller, & Landerl, 2014; Vorstius, Radach, & Lonigan, 2014).

The grade-appropriate information obtained from school textbooks is a further advantage for a children’s database, since it is relevant for capturing processing changes over time, such as the transition from the use of sublexical mapping (e.g., grapheme–phoneme correspondences) to more lexically based processing as children acquire reading skills (e.g., Rau et al., 2014), or the later-emerging automatic and coarse-grained orthography-based mechanisms that become established due to self-teaching (Share, 2004) and reading experience (e.g., Ziegler, Bertrand, Lété, & Grainger, 2014). As well as being vital for work on typical development, both within (e.g., Pattamadilok, Morais, De Vylder, Ventura, & Kolinsky, 2009; Ziegler et al., 2014) and across (e.g., Duncan, Casalis, & Colé, 2009; Duncan et al., 2013) different native languages, the control over the lexical characteristics of stimuli that children’s databases offer also strengthens research on second language learning (e.g., Commissaire, Duncan, & Casalis, 2014) and on developmental disorders such as dyslexia (e.g., Quémart & Casalis, 2015; Ziegler & Muneaux, 2007).

Some of the earliest examples of children’s databases were for American English, such as the *American Heritage Word Frequency Book* (Carroll, Davies, & Richman, 1971) and the *Educator’s Word Frequency Count* (Zeno, Ivens, Millard, & Duvvuri, 1995). The latter book is based on a 17-million-word corpus for 6- to 12-year-olds and has been found to be a very good predictor of lexical decision and naming RTs with young and older adults; indeed, it outperformed two widely used adult databases, the Kučera–Francis (1967) norms and CELEX (Balota et al., 2004).

Written-word frequency databases for children now exist in a number of European languages. For 5- to-9-year-old speakers of British English, there is the online Children’s Printed Word Database (CPWD), developed by Stuart, Dixon, Masterson, and Gray (2003) and extended by Masterson, Stuart, Dixon, and Lovejoy (2010). The updated database is compiled from the 1,011 reading books most commonly used by teachers during the first 4 years of schooling in a representative sample of UK primary schools. For French, there is the MANULEX database (Lété, Sprenger-Charolles, & Colé, 2004), which is grade-based (Grades 1 to 5), with a number of frequency indices that were computed from 1.9 million tokens. LEXIN (Corral, Ferrero, & Goikoetxea, 2009) is a Spanish psycholinguistic database for beginning readers focusing only on words from the 134 books used in kindergarten and first grade.

Two other European online databases for children have been constructed recently, which both contain measures of CD as well as the more traditional lexical information, such as frequency, part of speech (PoS), and orthographic form, that was included in previous databases. ESCOLEX (Soares et al. 2014) was developed for European Portuguese, compiled from 171 books for 6- to 11-year-old primary school children. In this database, CD is calculated as the proportion of textbooks in which the word appears, at any grade level. Preliminary investigation of this index indicated that CD is more explanatory of lexical decision times than word frequency among Grade 4 Portuguese speakers (Perea, Soares, & Comesana, 2013). The other database, childLex (Schroeder et al., 2015) is an age-based (6–12 years old), rather than a grade-based, German database computed from 500 books (almost 10 million tokens) read by children in their leisure time. ChildLex includes frequency, CD, word form, and lemma values, as well as orthographic Levenshtein distance (OLD20). The latter, which is also included in HelexKids, was first introduced by Yarkoni, Balota, and Yap (2008) and is based on the Levenshtein distance (LD) string metric. LD is defined as the minimum number of insertions, substitutions, and deletions required to generate one word from another. OLD20 is the mean number of the aforementioned alterations between a word and its 20 closest neighbors. OLD20 was found to predict lexical decision and naming latencies over and above Coltheart’s neighborhood size index (Coltheart, Davelaar, Jonasson, & Besner, 1977) in three different representative data sets (Yarkoni et al., 2008).

A further new addition is SUBLTEX-UK (van Heuven et al., 2014), an online database that consists of almost 202 million words (332,987 different word types) and contains values for CD (number and percentage of words appearing in 45,099 different television programs), PoS and dominant PoS frequency, lemma frequency, and Zipf frequency (a standardized logarithmic scale of frequency values; see the Indices section). Importantly, SUBTLEX-UK is the first psycholinguistic database to contain word forms from the subtitles appearing on children’s television broadcast channels—specifically, the UK channels *CBBC* and *CBeebies*. As part of the corresponding adult database, the CBBC and CBeebies frequency measures show the frequency trajectory from childhood to adulthood. Although it is not possible to obtain figures for specific age ranges from SUBLTEX-UK, the Zipf frequency values have a good overall correlation with the written log frequencies in CPWD (*r* = .756 for Cbeebies, and *r* = .690 with CBBC).

### Psycholinguistic databases for the Greek language

Three databases currently exist for use in language research with Greek-speaking adult participants: the HNC (2009), developed by the Institute of Language and Speech Processing in Athens, Greece; GreekLex (Ktori, van Heuven, & Pitchford, 2008) as well as GreekLex 2 (Kyparissiadis, van Heuven, Pitchford, & Ledgway, 2015); and SUBTLEX-GR (Dimitropoulou et al., 2010).

The HNC, available at http://hnc.ilsp.gr, is a Modern Greek written-word form and lemma frequency database that currently contains 47 million words and is continually updated. Words are extracted mainly from newspapers (61%), books (9%), magazines (6%), and other miscellaneous sources (23%; leaflets, brochures, etc.). Frequency information from the HNC corpus is freely available from the website, but for full access to all subcorpora and to lemma and PoS values, a subscription is required.

GreekLex contains 35,304 different word types that were all entries in both a Lexicon of Common Modern Greek (Aristotle University of Thessaloniki, 1998) and in the HNC. The database provides values for word form and lemma frequency, number of orthographic neighbors (substitution, transposition, addition, and deletion), neighborhood frequency, and letter and bigram frequencies. The database can be downloaded from www.psychology.nottingham.ac.uk/GreekLex. GreekLex 2 will be an upgrade of GreekLex with new variables included, such as phonological neighborhood size and PoS frequencies.

Finally, SUBTLEX-GR (available at www.bcbl.eu/databases/subtlex-gr/) was compiled from over 27 million words extracted mainly from subtitled American-English movies and TV series. It provides values for frequency, number of orthographic neighbors (substitution), word length, OLD20, and CD. Comparison with the text-based GreekLex database indicates that the SUBTLEX-GR frequency values are more explanatory in regression analyses, showing an advantage of more than 10% over the GreekLex values in predicting adult lexical decision performance (Dimitropoulou et al., 2010). Until now, no developmental database has been constructed for Greek, which is surprising, given the interest in studying typical and atypical literacy development in Greek (e.g., Douklias, Masterson, & Hanley, 2008; Harris & Giannouli, 1999; Loizidou-Ieridou, Masterson, & Hanley, 2010; Nikolopoulos, Goulandris, Hulme, & Snowling, 2006; Niolaki & Masterson, 2013; Niolaki, Masterson, & Terzopoulos, 2014; Niolaki, Terzopoulos, & Masterson, 2014; Porpodas, 1999) and the considerable amount of cross-linguistic research that involves Greek children (Dimitropoulou, Duñabeitia, & Carreiras, 2011; Duncan et al., 2013; Goswami, Porpodas, & Wheelwright, 1997; Ktori & Pitchford, 2008; Niolaki & Masterson, 2013; Seymour, Aro, & Erskine, 2003). The enduring interest in cross-linguistic comparisons with Greek can be attributed to the fact that, although it is an alphabetic language, Greek offers an orthographic contrast to other European languages at the letter symbol level (see Dimitropoulou et al., 2011). Another distinctive aspect of Greek is that it is considered a transparent language (Seymour et al., 2003), with consistent feedforward mappings (from orthography to phonology), but less consistent mappings in the feedback direction (Protopapas & Vlahou, 2009), particularly for certain vowels.

Due to the shallow orthography, Greek developing readers may rely more on sublexical processing using small grain-size units (grapheme–phoneme level) while reading (Ziegler & Goswami, 2005). The high transparency means that typical and atypical reading acquisition is investigated more often via speeded measures such as reading fluency (Protopapas, 2016), which leads to a demand for precise indices of frequency and orthographic form. However, the fact that there is feedback inconsistency may be addressed by the child by placing more reliance on larger sublexical units (e.g., syllables) or on lexical processing involving whole-word representations. Indeed, spelling appears more difficult than reading for Greek (Niolaki & Masterson, 2013), and Niolaki, Masterson, and Terzopoulos (2013) found that older children (9 years old and above) rely to a greater extent than younger children on whole-word orthographic processing, as their spelling performance is associated with visual attention span. Because sight vocabulary size is related to reading experience (Stanovich & Cunningham, 1992), the more frequently a word occurs and the more varied the contexts in which the word occurs, the stronger these representations will be.

Although it has proved possible to estimate frequency on an individual-school basis by sampling stimuli from the classroom experience of Greek beginning readers (e.g., Duncan et al., 2013), as children’s reading experience increases, more evident is the need for a developmental psycholinguistic database to inform within-language investigations and cross-linguistic comparisons. Up to now, in most studies with Greek-speaking children, for which tests were not available in Greek or a set of experimental Greek stimuli had to be selected, the corresponding English tests were translated, or the stimuli were taken from adult databases. Unfortunately, pragmatic solutions, such as translating tests without validating them for reliability and validity or selecting stimuli from databases that are not age-appropriate, introduce uncertainty about the status of existing findings, further highlighting the need for a developmental database in Greek.

### The HelexKids database

HelexKids fills this gap in Greek by providing written frequency values from school textbooks for children between 6 and 12 years old. HelexKids, although not as large as Brysbaert and New (2009) have advocated, is substantial in size, at 1.3 million words, and is likely to provide accurate objective frequency values for the school population in Greece and Cyprus, where the same textbooks are used in all primary schools. The Greek (and Cypriot) national curriculum is mandatory for the six grades of primary school, and every child and teacher has to use the authorized books. These are the main reference in each lesson, and they are used for reading, spelling, writing, memorizing, practicing, problem-solving, and assessment. Since these textbooks are used by the whole school population, they were considered to be a representative corpus, particularly because children are required to read them not only at school, but also at home. In the Greek and Cypriot educational systems, pupils take their textbooks home, as they have a relatively large amount of daily homework based on them. An important advantage of using the textbooks is that they provide a very precise way of looking at development across different grades, and using the textbooks also means that the database reflects the experience of both Greek and Greek-Cypriot children, whereas it is not clear that their fictional reading experiences would be similar. Moreover, there are not national statistics on the titles of fictional books that are possibly most bought or borrowed by children, which would make the selection of appropriate fiction materials problematic. It is therefore assumed that the corpus, despite the limited number of observations per grade, reflects the actual reading experience of the language users, and that there is little variation between users regarding the amount and age of exposure to the printed words that should constitute their core reading vocabulary.

On the basis of this corpus, HelexKids provides the Zipf standardized frequency value along with three other frequency indices: dispersion (D), estimated frequency per million (U), and the standard frequency index (SFI). HelexKids also includes CD and OLD20 values. All of these measures are described in the Indices section.

The decision was taken to include figures for CD and orthographic similarity alongside the frequency values. Our reasoning was threefold: First, these measures would allow researchers using HelexKids to access a wider range of psycholinguistic variables in existing databases for comparative research (e.g., when contrasting child and adult performance in Greek or when making cross-linguistic comparisons of young readers); second, CD has been shown to be the best predictor of lexical decision latencies in Greek (Dimitropoulou et al., 2010); and third, further investigation of the underresearched question of orthographic neighborhood effects in Greek is overdue.

### Corpus sampling

HelexKids contains words from 116 books used across the six grades of primary education in Greece and Cyprus. All of the books that are used in primary education in Greece and Cyprus are free and available online to all children and teachers at http://dschool.edu.gr/. The books were created by interdisciplinary groups (university lecturers, researchers, teachers, and writers), and they are rooted in recent theories of education. The most recent update of the textbooks happened progressively between 2007 and 2013.

Greek and Cypriot primary education starts when children are 6 years old (Grade 1) and extends until they are 12 years old (Grade 6). The Greek national curriculum is compulsory for all pupils, and the main subjects taught are Greek language, foreign languages (English in all grades and French or German for Grades 5 and 6), mathematics, environmental studies, science, Greek history, religious education, art, musical education, physical education, geography, citizenship, and theatre. Foreign-language textbooks were not included in HelexKids because it is a Greek-only database, although loans from other languages (e.g., “goal” /γκολ/ and “computer” /κομπιούτερ/) were not excluded, as they are part of children’s typical spoken and written vocabulary. Table 1 presents the numbers of textbooks in each grade per subject area. It should be noted here that some books are used in more than one grade. These books are included in the grades that they were written for (e.g., the Greek grammar book is included in both Grades 5 and 6), but in the total lexicon (Grades 1 to 6) they are included only once.

**Table 1:** Numbers of different textbooks in each grade, tabulated by school subject

Subject	Grade 1	Grade 2	Grade 3	Grade 4	Grade 5	Grade 6	All Grades^*^
Greek	5	6	6	7	8	8	34
Mathematics	6	6	5	5	5	5	32
Environmental studies	2	2	2	2	0	0	8
Science	0	0	0	0	2	2	4
History	0	0	2	2	2	2	8
Geography	0	0	0	0	2	2	4
Religious education	0	0	1	1	1	1	4
Music education	2	2	2	2	2	2	10
Art	2	2	2	2	2	2	6
Theatre	0	0	0	0	1	1	1
Physical education	1	1	1	1	1	1	3
Citizenship	0	0	0	0	1	1	2
TOTAL	18	19	21	22	27	27	116

Books used in more than one grade contribute only once to the total number.

**Fig. 1:**
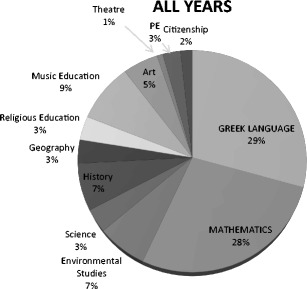
Distribution of textbooks per subject

**Fig. 2:**
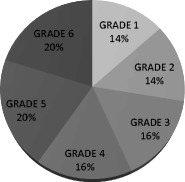
Distribution of textbooks per grade

From Table 1, it is apparent that the total number of books increases from grade to grade, with Grade 1 having the fewest books and Grades 5 and 6 the most (this is in accordance with the number of tokens; see Table 2). The difference in the number of books used between Grade 1 and Grade 5 or 6 (i.e., nine textbooks = 7.8%) can be attributed to the fact that new subjects (science, geography, theatre, and citizenship) are introduced in Grade 5. In total, the most books are used for teaching Greek (*M* = 6.66) and mathematics (*M* = 5.33). Figures 1 and 2 present the total percentages of books per subject and per grade in relation to the total number of 116 textbooks.

**Table 2:** Numbers of tokens for each grade by subject

Subject	Grade 1	Grade 2	Grade 3	Grade 4	Grade 5	Grade 6
Art	4,881	4,881	12,465	13,676	13,104	13,104
Citizenship					9,766	18,451
Environmental studies	7,093	11,303	20,560	46,062		
Geography					33,317	29,399
Greek	38,092	48,972	79,391	151,170	206,695	208,906
History			51,146	37,266	38,931	51,759
Mathematics	12,920	22,744	17,684	23,470	27,324	76,162
Music	2,317	2,732	10,726	10,725	18,550	26,927
Religious education			22,629	43,008	32,557	23,192
Science					40,916	42,940
Theatre					16,926	16,926
TOTAL	70,352	95,681	226,932	337,708	462,682	532,362

All textbooks were manually downloaded and then proofread. First, they were cleared of punctuation, hyphens, numbers, symbols (e.g., %), isolated syllables and letters, non-Greek letters and words, abbreviations (e.g., μμ [pm]), acronyms (e.g., ΕΕ [EU]), and names and surnames (e.g., of authors). All pages with tables of contents, introductory notes, and bibliographies designed only for teachers’ consideration were excluded. In contrast, words in capital letters (e.g., in titles or headers) or starting with a capital letter were included, as these were considered an integral part of children’s reading experience. Hyphenated words were not included, as these are rare in Greek. The database contains all words with inflections (e.g., plurals). Proper nouns (e.g., “Athens”) and proper names (e.g., “Alexander the Great”) were kept in the database because they constitute essential vocabulary, particularly in primary education. The database was then cleared of any spelling and stress mistakes with the aid of a conventional spell checker and of more specialized software, Symfonia, developed by the Institute of Language and Speech Processing in Greece. The spelling errors, mainly found in the Greek-language textbooks, were made on purpose by their authors as part of spelling exercises in which children were expected to find the mistake and correct it.

### Indices

HelexKids contains the following frequency indices and lexical variables: *F*, the raw frequency count; *F/m*, the frequency per million; *logF/m*, the logarithmic transformation of F/m; *Zipf*, a standardized frequency value recently introduced by van Heuven et al. (2014); *D*, the dispersion of occurrences between textbooks; *U*, the frequency per million adjusted in relation to the dispersion index; *SFI*, a standard frequency index; *CD*, contextual diversity; *Nletters*, the number of letters; and *OLD20*, a measure of orthographic Levenshtein distance. The grade and the cumulative lexicons contain all values apart from Zipf and OLD20, which were calculated for the all-grades lexicon only. Each of the indices is described in turn below.

#### Zipf

Zipf is a standardized frequency value expressed on a logarithmic scale, first introduced by van Heuven et al. (2014) and then used by Soares et al. (2015) in the SUBTLEX-PT database. The Zipf scale ranges from 1 to 6: Words with a Zipf value of 1 are considered to be of very low frequency (1 per 100 million words), those with a value of 6 to be very high-frequency (1 per 1,000 words), and those with a value between 3 (1 per million words) and 4 (1 per 100,000 words) to be of medium frequency. The Zipf scale, apart from the obvious advantage that it is relatively straightforward to use, also provides researchers with the ability to select items with a frequency below 1 per million that otherwise would have been excluded. Since the corpus is not large, we decided to calculate Zipf as follows:

$$ \mathrm{Zipf} = { \log}_{10}\left(\frac{raw\kern0.5em  frequency+1}{1.35+0.07}\right)+3 $$

The denominator corresponds to the number of tokens plus the number of word types. This equation captures the possibility that some words may have zero frequency because they happen to be unobserved, something that can particularly happen in corpora that are not very large, with most of the words being at the lower end of the frequency continuum (see Table 3). Thus, with this transformation the corpus size is considered to be larger than the actual size, by adding to it the number of different words. Thus, an unobserved word has a Zipf value of 2.84, a word with a frequency of 1 has a Zipf value of 3.25, and a word with a frequency of 10 has a Zipf value of 4.03. The values for the unobserved items are elevated because of the small size of the corpus, and they reflect the likelihood that words with zero frequency are not unknown by the students.

**Table 3:** Number of different word types, number of words occurring five or more times per grade, and number and percentage of hapax words, tabulated by grade

Grade	Word Types	Hapax Words	% of Hapax Words	Words Occurring 5 or More Times
Grade 1	9,155	4,533	49.5	1,889
Grade 2	11,714	5,791	49.5	2,453
Grade 3	21,193	9,373	44.4	5,172
Grade 4	33,762	16,267	48.2	7,511
Grade 5	44,851	21,396	47.7	9,797
Grade 6	48,080	22,641	47.1	10,881

- *Frequency count (F)* This is the number of occurrences of a word in the corpus.
- *Frequency per million (F/m)* This measure is calculated as F * 1,000,000/number of tokens.
- *Log frequency per million (logF/m)* is the log_10_ (F*/*m) + 1 (Laplace transformation). Adding 1 ensures that the logarithm of low-frequency items is not zero or negative (for items with F*/*m < 1).
- *Dispersion (D)* The dispersion of words across textbooks for each grade and for all grades is computed from the formula

$$ \mathrm{D} = \left\{ \log \varSigma \left({p}_{\mathrm{I}}\right)\hbox{--} \left[\left(\varSigma {p}_{\mathrm{i}} \log {p}_{\mathrm{i}}\right)/\varSigma {p}_{\mathrm{i}}\right]\right\}/{ \log}_{(n)}, $$

where *i* is the number of textbooks in which a word occurs, *p* is the probability of a word occurrence in textbook *i*, *n* is the number of textbooks in each grade, and Σ*p*_i_log *p*_i_ equals 0 when *p*_i_ = 0. When D = 1, the word appears in the same proportion in all textbooks, and when D = 0, the word appears in only one textbook.

#### Estimated frequency per million (U)

This index derives from the F/m index but is adjusted in light of D. If D = 1, then U is the frequency per million, and when D < 1, U is adjusted downward. If D = 0, then U has a minimum value based on the average weighted probability of that word in the 116 textbooks (Breland, 1996). The formula for calculating U is

$$ \mathrm{U} = \left(1,000,000/N\right)\left\{\mathrm{F}\mathrm{D} + \left(1\ \hbox{--}\ \mathrm{D}\right){f}_{\min}\right\} $$

*N* is the number of tokens, D is the index of dispersion, F is the frequency, and *f*_min_ = 1/*N* * Σ*f*_*i*_ *s*_*i*_ where *f*_*i*_ is the frequency of a word in textbook *i*, and *s*_*i*_ is the number of tokens in textbook *i*.

#### Standard frequency index (SFI)

This is an index derived from U and is calculated using the formula 10(log_10_ U + 4). SFI provides the researcher with an index that is easily understood: SFI = 90 means that a word occurs once every ten tokens; SFI = 80, once every 100 tokens; SFI = 70, once every 1,000 tokens; and so on. SFI is 0 when a word does not occur in the corpus.

#### Contextual diversity (CD)

This is an index of the occurrences of a word in different textbooks for each grade and for all grades. It is simply calculated as the number of textbooks in which a word appears in a grade, divided by the total number of textbooks for that grade. It equals 1 if the word appears in all textbooks, and its value ranges between .1 and 1. CD does not take account of absolute frequencies as D does, but it indicates instead how words are distributed in different contexts. We assume that each textbook corresponds to a particular context; for instance, the mathematics textbooks are contextually different from the music textbooks (see also Steyvers & Malmberg, 2003). In contrast, D provides the proportion of frequency occurrences across textbooks.

An example illustrating the differences between the indices above are the words έθιμο /ethimo/ “custom” and εκδήλωσης /ekthilosis/ “event,” which have the same frequency (Zipf = 4.35), the same CD (0.24), but different D values (.42 and .29, respectively). This is because, although both appear in five different textbooks, their frequency dispersions are different (1 + 1 + 4 + 10 + 4 and 2 + 1 + 15 + 1 + 1, respectively), and consequently, they have different U (21.09 and 14.28) and SFI (53.24 and 51.55) values. Thus, whereas CD and D correlate strongly (in HelexKids, the Pearson correlation coefficient is .77, *p* < .001), these indices are not interchangeable (see also Soares et al., 2015).

#### Number of letters (Nletters)

HelexKids provides the number of letters, ranging from 1 (18 different words) to 22 (two different words). The mean length is 8.75 (*SD* = 2.58), and eight-letter words are the most frequent, with 10,247 occurrences, or 14.9% of the total corpus of 68,692 types. Greek is a morphologically rich language in which derivational and inflectional morphology plays an important role, and words tend to be multisyllabic rather than monosyllabic in nature.

#### OLD20

This is the orthographic Levenshtein distance score (Yarkoni et al., 2008). For the calculation of OLD20 (all words in lowercase) a relevant R package was used, developed by Keuleers (2013).

### Textbooks and word statistics

In total, the tokens obtained from the 116 textbooks numbered 1,355,265. Table 2 presents the numbers of tokens for each grade by subject.

The most tokens come from Grades 5 and 6, with the least tokens occurring in Grade 1, which is in accordance with the number of textbooks in these grades. The different word types yielded gave a total of 68,692 tokens that occur across all grades. It is apparent from Table 2 that substantial increases in tokens take place between Grade 2 and Grade 3 (131,987 words), Grade 3 and Grade 4 (110,914), and Grade 4 and Grade 5 (124,767). The increase is less between Grade 5 and Grade 6 (69,133), and considerably less between Grade 1 and Grade 2 (25,364).

Table 3 presents the numbers of different word types, the numbers of words appearing five or more times per grade, and the numbers and percentages of hapax words (words that appear only once). A striking finding is the large percentage of hapax words in each grade, which suggests that almost 50% of the vocabulary that children encounter in print consists of words that occur only once. Furthermore, words occurring less than five times make up approximately 30% of the vocabulary. This indicates that a significant part of the growth in the size of the printed vocabulary, introduced as different word types in each grade, is the result of an increase in hapax words. However, since many of the hapax words are actually inflected forms of the same lemmas, they may still have strong connections to semantics, which may ease integration into the child’s sight vocabulary. Besides, Ehri (2005) suggests that only a few reading experiences are necessary for a word to become familiar. Nevertheless, for those hapax words that do not belong to a morphological family, their limited number and context of occurrences may impede the development of strong links to conceptual memory.

HelexKids is relatively small in comparison to other children’s databases in terms of the numbers of tokens and textbooks. Despite the smaller corpus from which it is computed, the number of word entries does not differ markedly from the other databases. For example, HelexKids has more different word types in Grade 1 (9,155) than ESCOLEX (8,316), although slightly less than MANULEX (11,331). This may be attributed to the large number of hapax words (e.g., 4,533 in HelexKids vs. 2,989 in ESCOLEX for Grade 1), which seems likely to reflect the fact that Greek is an extremely rich inflectional language. Regarding the number of textbooks, HelexKids is computed from considerably fewer books than childLex (500 books), and slightly fewer than ESCOLEX (172 books), but more than MANULEX (50 readers). All of the books, however, from which words were extracted in HelexKids are school textbooks, which is similar only to ESCOLEX. The differing compositions of the databases derive from differences in the educational systems: In Greece and Cyprus, as in Portugal, there is one national curriculum, and the same textbooks are used across all schools. Words extracted from these books should arguably be a valid index of the print that children are exposed to, since reading these books is part a compulsory program.

HelexKids also provides frequency values that represent cumulative experience (i.e., in Grades 1–2, 1–3, 1–4, 1–5, and 1–6; see also Martinez & Garcia, 2008). Frequency values in these lexicons correspond to the sum of the occurrences of a word that a child of a certain grade has been exposed to in all previous grades. For example, a Grade 4 child will have been exposed to a word not only in Grade 4, but also potentially during Grades 1, 2, and 3. A similar approach has been adopted in MANULEX for Grades 1–3 and 1–5, and in ESCOLEX for Grades 1–4, 5–6, and 1–6. Table 4 presents the numbers of tokens, word types, and hapax words for each cumulative lexicon.

**Table 4:** Number of tokens, of different word types, number of words occurring five or more times, and number and percentage of hapax words, tabulated per grade combination

Grade	Tokens	Word Types	Hapax Words	% of Hapax Words	Words Occurring 5 or More Times
Grades 1–2	165,864	13,531	4,353	32.2	3,798
Grades 1–3	391,731	26,338	9,400	35.7	8,661
Grades 1–4	729,363	41,648	14,301	34.3	13,557
Grades 1–5	1,191,971	59,402	18,464	31.1	19,640
All Grades	1,355,265	68,692	27,733	40.4	20,392

Comparing HelexKids to the existing Greek adult databases (SUBTLEX-GR and GreekLex) shows that there is a substantial difference in the number of word types relative to SUBTLEX-GR (145,361 entries), but not to GreekLex (35,304 entries). A further comparison between the variables these databases include reveals differences in mean word length, frequency per million, CD, and OLD20. In particular, the mean lengths in both adult databases are over nine letters (9.55 in SUBTLEX-GR, 9.14 in GreekLex), whereas in HelexKids (total lexicon) it is 8.75, suggesting that thewords in children’s textbooks are somewhat shorter than in adult-based texts or subtitle corpora. The mean frequency per million is considerably larger in HelexKids (15 occurrences per million, vs. 6.87 in SUBTLEX-GR and 4.29 in GreekLex), which indicates that more words in children’s textbooks have high or very high frequency in comparison to the adult databases. For example, in SUBTLEX-GR, just 0.9% (1,311) of the word types have a frequency per million value over 60, whereas in HelexKids the percentage is 2.5% (1,728 word types). In terms of CD, HelexKids has a mean value of just .05, whereas in SUBTLEX-GR (GreekLex does not provide CD values) the statistic is .81. This indicates that a large number of words in children’s textbooks appear only in specific textbooks (e.g., words appearing only in math books). Finally, in terms of orthographic similarity, the difference in OLD20 values between SUBTLEX-GR and HelexKids is not large (2.86 vs. 3.13, respectively), which indicates no significant changes in neighborhood size from childhood to adulthood, possibly due to the morphologically rich nature of the Greek language.

Finally, lemmatization was also conducted for the total lexicon (21,193 lemmas), by comparing the database with the lemma entries of GreekLex and the HNC. The reason for lemmatizing is that Greek is a rich inflectional language, and verbs, nouns, and adjectives (the most common grammatical categories in the HNC; Hatzigeorgiou, Mikros, & Carayannis, 2001) are found in many different inflectional forms. For example, verbs take six different suffixes, depending on the personal pronoun (I, you, he, etc.) and the tense (12 tenses exist for the active voice and 12 for the passive voice, all with different inflections). Nouns and adjectives are inflected for three different genders, two numbers (singular and plural), and four cases for each number. Although children may be exposed to many different inflectional forms of a word, they all derive from the same lemma. Therefore, we considered that lemma frequencies might provide a more accurate reflection of the lexico-semantic properties of words, since at the semantic level the same representation is activated by each inflected form. It must be noted here that some lemmas may change form when they are combined with different suffixes (e.g., the verb παίρνω /perno/ “take,” which corresponds to the first person in the simple present tense, becomes πήρα /pira/ “took” in the simple past tense). Although the two forms may have different orthographic codings, they belong to the same family semantically.

It is obvious from the “G1–G6” column in Table 5 that the mean frequency across grades is low (only in Grades 1 and 2 are the mean Zipf values just above 4), and that the most common frequency value is 1. This suggests that most words are not encountered frequently by the children, which may hamper vocabulary acquisition, spelling performance, and reading speed. Frequencies decrease as grade increases, as is also shown by the U values, suggesting that an increase in the number of textbooks and printed vocabulary size (see Tables 1 and 2) is not accompanied by an increase in mean frequencies. In fact, only 16% of the words have a Zipf value over 4, and the total distribution shows a clear bias toward low-frequency words (82.9% have a Zipf value between 3 and 4—i.e., words with raw frequencies between 1 and 9). On the other hand, the 100 most frequent words account for 44.9% of the total tokens, which indicates that this small number of words are the ones most commonly used in Greek school books. This suggests that the most frequent words represent only a very small proportion of the different word types (just 0.007% across all grades).

**Table 5:** Mean, mode, minimum, maximum, and percentiles values (P10, P25, P50, P75, and P90) for all grades for the frequency counts, Zipf, D, U, SFI, and CD

		G1	G2	G3	G4	G5	G6	G1–G6
Frequency	Mean	7.68	8.15	10.68	10	10.30	11.07	19.67
	Mode	1	1	1	1	1	1	1
	Minimum	1	1	1	1	1	1	1
	Maximum	2,912	3,462	8,604	11,220	14,967	17,481	46,576
	P10	1	1	1	1	1	1	1
	P25	1	1	1	1	1	1	1
	P50	2	2	2	2	2	2	2
	P75	4	4	4	4	4	4	6
	P90	10	10	12	11	11	11	19
Zipf	Mean	4.25	4.15	3.90	3.78	3.71	3.69	3.62
	Mode	4.18	4.06	3.73	4	4	3.47	3.25
	Minimum	4.18	4.06	3.73	4	4	3.47	3.25
	Maximum	6.47	6.54	6.93	7	7	7.24	7.67
	P10	4.18	4.06	3.73	3.60	3.51	3.47	3.25
	P25	4.18	4.06	3.73	3.60	3.51	3.47	3.25
	P50	4.21	4.10	3.81	3.70	3.62	3.59	3.45
	P75	4.26	4.16	3.93	3.84	3.79	3.77	3.83
	P90	4.39	4.31	4.22	4.15	4.12	4.11	4.30
D	Mean	0.14	0.15	0.15	0.14	0.14	0.14	0.16
	Mode	0	0	0	0	0	0	0
	Minimum	0	0	0	0	0	0	0
	Maximum	0.95	0.97	0.95	0.96	1	0.95	0.95
	P10	0	0	0	0	0	0	0
	P25	0	0	0	0	0	0	0
	P50	0	0	0	0	0	0	0.12
	P75	0.29	0.30	0.26	0.25	0.23	0.23	0.27
	P90	0.48	0.51	0.49	0.48	0.46	0.46	0.47
U	Mean	71.02	57.54	31.89	20.04	15.16	14.72	10.28
	Mode	3.92	2.12	0.59	0.43	0	0.17	0.03
	Minimum	0.47	0.27	0.10	0.44	0	0.01	0
	Maximum	38,357	33,649	34,968	30,666	30,060	30,699	31,745
	P10	0.87	0.92	0.29	0.23	0.11	0.08	0.01
	P25	1.86	1.26	0.59	0.38	0.16	0.17	0.02
	P50	3.92	2.12	1.18	0.51	0.30	0.25	0.24
	P75	18.52	13.18	6.27	3.73	2.53	2.25	1.16
	P90	67.86	56.19	25.92	15.14	10.47	9.67	6.02
SFI	Mean	48.12	46.70	42.95	40.66	38.44	37.76	32.51
	Mode	45.94	43.26	37.72	36.35	34	32.40	24.15
	Minimum	36.70	34.34	29.97	26.46	23	20.73	10.87
	Maximum	85.84	85.27	85.44	84.87	85	84.87	85.02
	P10	39.40	39.65	34.67	33.55	30.60	29.05	20.45
	P25	42.69	40.99	37.72	35.76	32.16	32.32	23.34
	P50	45.94	43.26	40.73	37.12	34.83	33.90	33.85
	P75	52.68	51.20	47.98	45.72	44.03	43.51	40.66
	P90	58.32	57.50	54.14	51.80	50.20	49.86	47.80
CD	Mean	0.18	0.19	0.14	0.13	0.11	0.11	0.05
	Mode	0.10	0.10	0.07	0.06	0	0.05	0.01
	Minimum	0.10	0.10	0.07	0	0	0.05	0
	Maximum	1	1	1	1	1	1	0.99
	P10	0.10	0.10	0.07	0.06	0.05	0.05	0.01
	P25	0.10	0.10	0.07	0.06	0.05	0.05	0.01
	P50	0.10	0.10	0.07	0.06	0.05	0.05	0.03
	P75	0.20	0.20	0.13	0.13	0.10	0.10	0.05
	P90	0.40	0.40	0.33	0.25	0.24	0.24	0.12

Similarly, D is also low, ranging on average between .14 and .16 in all grades, which in practice means that the average percentage of textbooks per grade in which a word appears is below 16. In addition, the possibility of encountering a word in different contexts, as measured by CD, is also low, on average only 5% for all grades (i.e., six books out of 116), ranging from 11% in Grades 5 and 6 to 19% in Grade 2. The low CD suggests that the majority of words are context-specific. SFI, on the other hand, is more normally distributed (see also Soares et al., 2015), with a mean of 32.51 and a median of 33.85 for Grades 1 to 6. The corresponding values for each grade are 48.12 and 45.94 for Grade 1, 46.70 and 43.26 for Grade 2, 42.95 and 40.73 for Grade 3, 40.66 and 37.11 for Grade 4, 38.44 and 34.83 for Grade 5, and 37.76 and 33.90 for Grade 6, respectively. Thus, SFI percentile values can be used as cutoff points when selecting low- and high-frequency words in each grade when Zipf values are not available, as in ESCOLEX and MANULEX.

### Availability of the HelexKids database website

The Web version of the HelexKids database is freely available at www.helexkids.org for searching and downloading content. The facility has been constructed using an online platform for website construction (www.manypage.com), which can incorporate programmable components such as the spreadsheet viewer and search filters used here.

The website contains 11 lexicons, one for each of the six grades and five cumulative lexicons (Grades 1–2, 1–3, 1–4, 1–5, and 1–6). Each lexicon consists of nine columns: the word spelling, frequency count (F), frequency per million (F/m), log frequency per million (logF/m), dispersion (D), estimated frequency per million (U), standard frequency index (SFI), contextual diversity (CD), and number of letters (Nletters). The “all grades” (1 to 6) lexicon has 11 columns, since Zipf and OLD20 values were also calculated. The user can search for the word variables of interest with the aid of nine filters. The first one is a letter filter that allows for searching for the letter or combination of letters that words should start with, end with, contain, or not contain. The other filters allow the user to search for specific values for Zipf, F, F/m, LogF/m, D, U, SFI, CD, and Nletters, with the aid of six functions: “=” (equals), “!=” (not equal to), “<” (less than), “<=” (less than or equal to), “>” (greater than), and “>=” (greater than or equal to).

In addition, the SELECT filter allows the user to obtain values for a particular word list. By pressing “change list” in the popup window, words can be typed or copied from another file and are returned (in the same order they were entered) with their corresponding values from the database. If other filters are also active, all words are returned, but only the selected values will be displayed. The returned words can be downloaded in comma-separated value (,csv) format. Finally, all database files are available for downloading from the website as Excel files.

## Conclusion

HelexKids is the first Greek word database for children. The database provides frequency values based on the printed vocabulary experienced by primary school children in Greece and Cyprus. It does so for each grade, offering the opportunity to obtain frequency trajectory values from Grade 1 (6 years old) to Grade 6 (12 years old). The database is a powerful tool for psycholinguists who are interested in literacy development and in theoretical and computational models of reading and writing. It provides researchers with new variables not previously available for Greek, such as Zipf, D, U, and SFI. It will therefore be a valuable resource in cross-linguistic studies and comparisons between languages for which similar databases exist.

Due to being based on curriculum textbooks, HelexKids will also be of great use to educational practitioners for designing appropriate instruction for pupils with Greek as a first or second language, and for teachers of students with special educational needs when constructing assessment tools and intervention programs. Finally, the database will be helpful for publishers and writers to consult before deciding on the lexical content of books and other media that are intended to be used by children from different age groups.

In the future, we plan to extend the HelexKids database to provide values for phonological neighbors, numbers of phonemes and syllables, PoS, and CV type. Moreover, further validation of the database will take place, to compare the variance accounted for by the grade-level and cumulative HelexKids frequency values in relation to children’s lexical decision latencies. The database will be updated as textbooks change and new editions or new books are introduced in primary education. We also intend to develop a Cypriot-only version of HelexKids, by including some textbooks that are used exclusively in Cyprus (e.g., for math and science). Additionally, a database for Greek with a combination of book and subtitle corpora would be very useful, as it could capture sufficiently not only the reading materials that children are exposed to at school and home, but also the large amount of exposure to Greek subtitles of foreign TV programs that they watch in their leisure time. Finally, HelexKids will provide the template for the development of HelexKids-bilinguals, a database for Greek–English bilingual children.

## Appendix A

**Table 6:** Textbooks used in more than one grade

Books	Grades 1 and 2	Grade 3 and 4	Grades 5 and 6	Grades 4, 5, and 6
Grammar			✓	
Dictionary				✓
Anthology of short stories and poems	✓	✓	✓	
Artistic expression	✓	✓	✓	
Music		✓		
Theatre			✓	
Physical education	✓	✓	✓	
